# Renal function and cognitive performance in older adults: a NHANES-based mediation analysis of methylmalonic acid as a marker of mitochondrial dysfunction

**DOI:** 10.1080/0886022X.2025.2577843

**Published:** 2025-11-17

**Authors:** Lijun Xia, Yong Zhang, Xiaoyang Lei, Xiaoying Ma, Ming Zhang, Dian He

**Affiliations:** a Department of Neurology, Affiliated Hospital of Guizhou Medical University, Guiyang, Guizhou, China; b Department of Neurology, Xingyi People’s Hospital, Xingyi, Guizhou, China; c Department of Imaging, Xingyi People’s Hospital, Xingyi, Guizhou, China; d Department of Neurology, Guihang Guiyang Hospital, Guiyang, Guizhou, China

**Keywords:** Cognitive impairment, methylmalonic acid, mitochondrial dysfunction, mediation analysis, NHANES, kidney function

## Abstract

Chronic kidney disease (CKD) is associated with cognitive impairment in older adults. Mitochondrial dysfunction (MD) is a key contributor to aging-related diseases, yet its potential role in mediating the relationship between renal function and cognitive function (CF) remains unclear. Therefore, we aimed to examine this association in older adults and to evaluate the mediating effect of MD. Our study analyzed data from the 2011–2014 National Health and Nutrition Examination Survey, including participants aged ≥60. CF was assessed using the Animal Fluency Test (AFT), the Consortium to Establish a Registry for Alzheimer’s Disease (CERAD) Word Learning Test, and the Digit Symbol Substitution Test (DSST). Multivariate linear regression models were used to explore the relationships between renal function and CF, CKD, and methylmalonic acid (MMA) levels, and CF. Mediation analysis was conducted to assess the role of MD, as reflected by serum MMA levels, in the link between renal function and cognitive impairment. A total of 2,437 participants aged ≥60 were included. Regression analysis showed a significant positive association between renal function and CF (β = 0.018, 95%CI: 0.013–0.024, *p* < 0.001). After adjusting for potential confounders, MMA levels mediated 13.33% of the relationship between renal function and CF. The indirect effect of renal function on CF *via* MMA was significant (β = 0.000949, 95%CI: 0.000419–0.002463, *p* = 0.002), as was the direct effect (β = 0.006165, 95%CI: 0.000271–0.010456, *p* = 0.034). This study identifies a potential link between CKD and CF in older US adults. MD, assessed by serum MMA levels, may partially mediate this relationship, with MMA potentially serving as a biomarker for cognitive impairment.

## Graphical abstract

**Figure:**
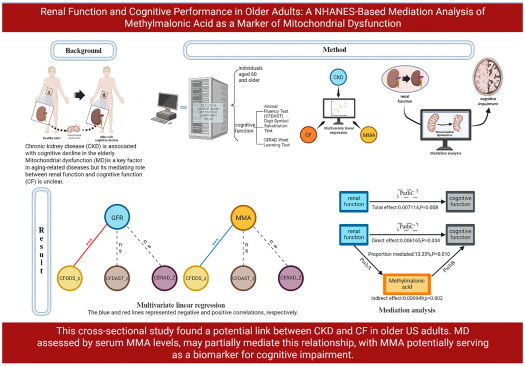

## Background

Chronic kidney disease (CKD) is a prevalent public health challenge impacting more than 800 million people or 10% of the population worldwide [1]. It is projected that the global incidence of CKD will increase rapidly by 2024, making it the fifth leading cause of years of life lost [2,3]. CKD refers to chronic structural and functional impairment of the kidneys caused by various etiologies, characterized by a persistent reduction in glomerular filtration rate (GFR) to <60 mL/min/1.73 m^2^ for more than three months [4,5], and is classified into five stages according to GFR [6].

Cognitive impairment is a syndrome involving global deficits in brain processes that affect learning, memory, and sensory information processing [7]. Patients with CKD are at increased risk for cognitive impairment, and this risk rises in parallel with kidney dysfunction [8,9]. CKD-related cognitive impairment tends to manifest as vascular cognitive impairment, predominantly characterized by marked deficits in executive function, attention, planning, and processing speed [10,11]. Compared with the general population or individuals without CKD, CKD patients have higher incidences of stroke [12], subcortical infarction, cerebral microbleeds, and white matter hyperintensities [12,13]. In CKD, the clearance of potential uremic toxins, such as neuropeptide Y (NPY), parathyroid hormone (PTH), and fibroblast growth factor 23 (FGF23), is reduced. Elevated circulating NPY levels may induce cerebral microvascular endothelial dysfunction and alterations of the blood-brain barrier (BBB), contributing to brain dysfunction. Increased PTH levels in CKD elevate cerebral alkaline phosphatase activity, which dephosphorylates tau protein. This facilitates the binding of tau to muscarinic receptors on hippocampal neurons, triggering calcium influx and neuronal apoptosis [7], ultimately leading to cognitive impairment.

Methylmalonic acid (MMA) is a mitochondrial intermediate metabolite generated during the catabolism of odd-chain fatty acids and certain amino acids (isoleucine, valine, methionine, and threonine). Under physiological conditions, these amino acids are converted to succinyl-CoA and enter the Krebs cycle [14]. MMA metabolism depends on intact mitochondrial function [15]. Mitochondrial dysfunction (MD) results in MMA accumulation [16]. Circulating MMA concentrations are markedly elevated in CKD compared to non-CKD populations, and MMA levels are significantly correlated with the degree of renal impairment in CKD patients [17]. An increasing body of evidence indicates that MMA plays a key role in MD and oxidative stress (OS) both *in vitro* and *in vivo* [18,19]. Significant differences in serum vitamin B12, folate, and homocysteine concentrations have been reported between CKD and non-CKD populations [20]. Metabolic vitamin B12 deficiency is common in CKD, and MMA or homocysteine serves as a functional biomarker of vitamin B12 status [21]. Notably, MMA may be a specific and sensitive biomarker of subclinical vitamin B12 deficiency [22]. Current evidence suggests an association among serum MMA, vitamin B12 status, and cognitive function (CF) in older adults [23]. MMA may impair cognition through the following mechanisms: (1) inhibition of the mitochondrial respiratory chain, reducing ATP production and disrupting neuronal energy metabolism; (2) induction of OS and neuroinflammation, accelerating neuronal apoptosis; and (3) interference with hippocampal synaptic plasticity [18,24,25]. Therefore, it is hypothesized that MMA mediates the relationship between renal function and CF, and that its association with poor cognitive and physical performance is stronger than that of serum vitamin B12 [26]. Based on this rationale, the present study aims to utilize data from the National Health and Nutrition Examination Survey (NHANES) to elucidate the association between renal function and CF, and to investigate the mediating role of MMA in this relationship.

## Methods

### Study design and population

**Figure 1.**
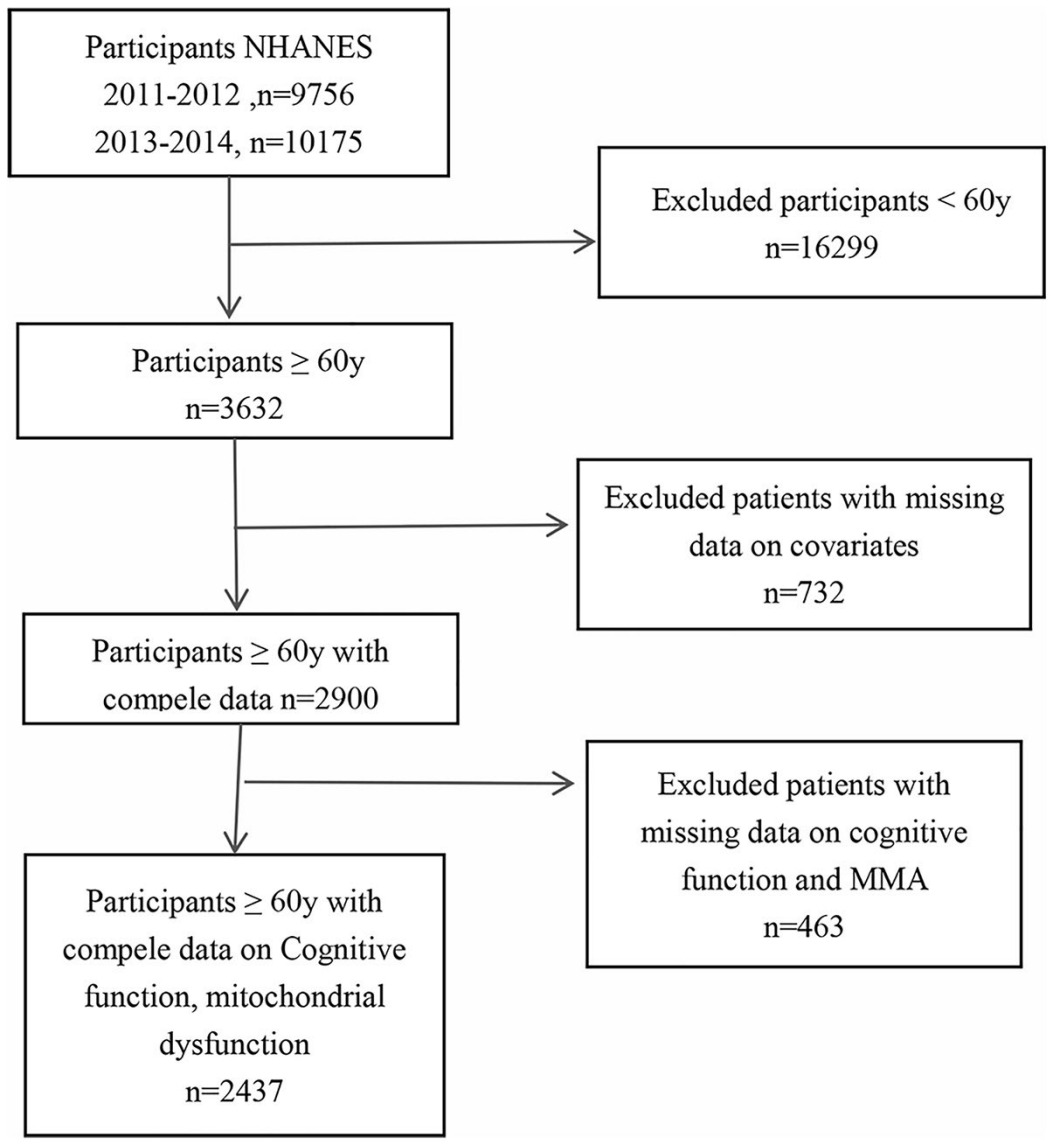
Flow diagram.

A cross-sectional study was undertaken based on 2011–2012 and 2013–2014 CKD and cognitive data from NHANES, which is a representative survey of Americans conducted biannually and encompasses health and nutrition data from a stratified, multistage probability sample of non-institutionalized civilians. The study’s data collection protocols received approval from the Research Ethics Review Board of the National Center for Health Statistics. All participants provided written informed consent. Because this analysis used publicly available NHANES data, an additional institutional ethics review was not required. Individuals younger than 60 years, those lacking CF measurements, and those with incomplete data on CKD or covariates were excluded from the final analytic sample (Figure 1).

### CKD assessment

The definition of CKD was based on the 2021 KDIGO guidelines (15). The estimated glomerular filtration rate (eGFR) was calculated using the CKD Epidemiology Collaboration (CKD-EPI) equation. Serum creatinine (sCr) was measured using a validated high-performance liquid chromatography (HPLC) method, and urinary albumin was quantified by solid-phase fluorescence immunoassay (FIA). For males, the calculation formula for eGFR was GFR = 142 × (Scr/0.9)^*a* × 0.9938^Ag. If Scr ≤ 0.9 mg/dL, a = −0.302; if Scr > 0.9 mg/dL, a = −1.200. For females, the calculation formula for eGFR was GFR = 142 × (Scr/0.7)^*a* × 0.9938^Age. If Scr ≤ 0.7 mg/dL, a = −0.241; If Scr > 0.7 mg/dL, a = −1.200.

### CF assessment

CF was evaluated in participants aged ≥ 60 years. Cognitive testing was conducted during the 2011–2014 NHANES cycles and included the Digit Symbol Substitution Test (DSST), the Animal Fluency Test (AFT), and assessments from the Consortium to Establish a Registry for Alzheimer’s Disease (CERAD). Higher scores indicated better cognitive performance. To facilitate comparison, all scores were standardized as z-scores.

1. CERAD Word Learning Test: This instrument assessed immediate and delayed recall of newly learned verbal material. It comprised three learning trials in which participants read aloud and then recalled as many words as possible from a list of 10. A delayed recall trial followed completion of the AFT and DSST. The immediate recall score (0–30) represented the total number of words recalled across the three learning trials; the delayed recall score (0–10) represented the number of words recalled after the delay.
2. AFT: This test assessed verbal fluency by requiring participants to name as many animals as possible within one minute. Each correct response was awarded one point, and the AFT score represented the total number of animals named.
3. DSST: A subtest of the Wechsler Adult Intelligence Scale, the DSST measured executive function and processing speed. Within two minutes, participants matched symbols to corresponding numbers according to a key. One point was awarded for each correct match, with a maximum possible score of 133.

### Mediator and covariate assessment

MMA was quantified *via* liquid chromatography-tandem mass spectrometry (LC-MS/MS) as the dibutyl ester derivative. MMA was extracted from 75 μL of serum, along with an internal standard (D3-MMA), using liquid-liquid extraction with tert-butyl methyl ether in an acidic medium. The resulting acid was derivatized with butanol to yield the dibutyl ester. Following vacuum evaporation of butanol, derivatized samples were reconstituted in acetonitrile/water. The preparation process required approximately 4 h and allowed for the simultaneous analysis of 96 samples (calibrators, quality controls, and unknowns). MMA quantification employed isocratic LC-MS/MS with a retention time of 5.9 min, ensuring separation from potential interferences such as isomeric succinic acid. Quantification was based on the peak area ratio relative to a six-point aqueous calibration curve.

Covariates were selected for their presumed link to renal function and cognitive impairment in older adults. These covariates included: 1) sociodemographic variables: age (years), sex (male or female), race/ethnicity (Mexican-American, non-Hispanic White, non-Hispanic Black, or others including multiracial), education (less than high school, high school diploma, or above high school diploma), poverty-to-income ratio (PIR) and marital status(married/cohabitating, widowed/divorced/separated, or never married); 2) behavioral factors: smoking (never/former/current smoker), alcohol consumption (≥12 or <12 drinks per year), physical activity, body mass index (BMI), sCr (mg/dL), and health conditions(diabetes and hypertension). Given prior evidence indicating that social support may influence cognitive health and access to medical care, marital status was incorporated as a covariate, as it has the potential to confound the relationship between kidney function and cognitive outcomes [6]. The NHANES database is publicly available and freely accessible; it has been approved by the Research Ethics Review Board of the National Center for Health Statistics. Written informed consent was obtained from all participants.

### Statistical analysis

**Figure 2.**
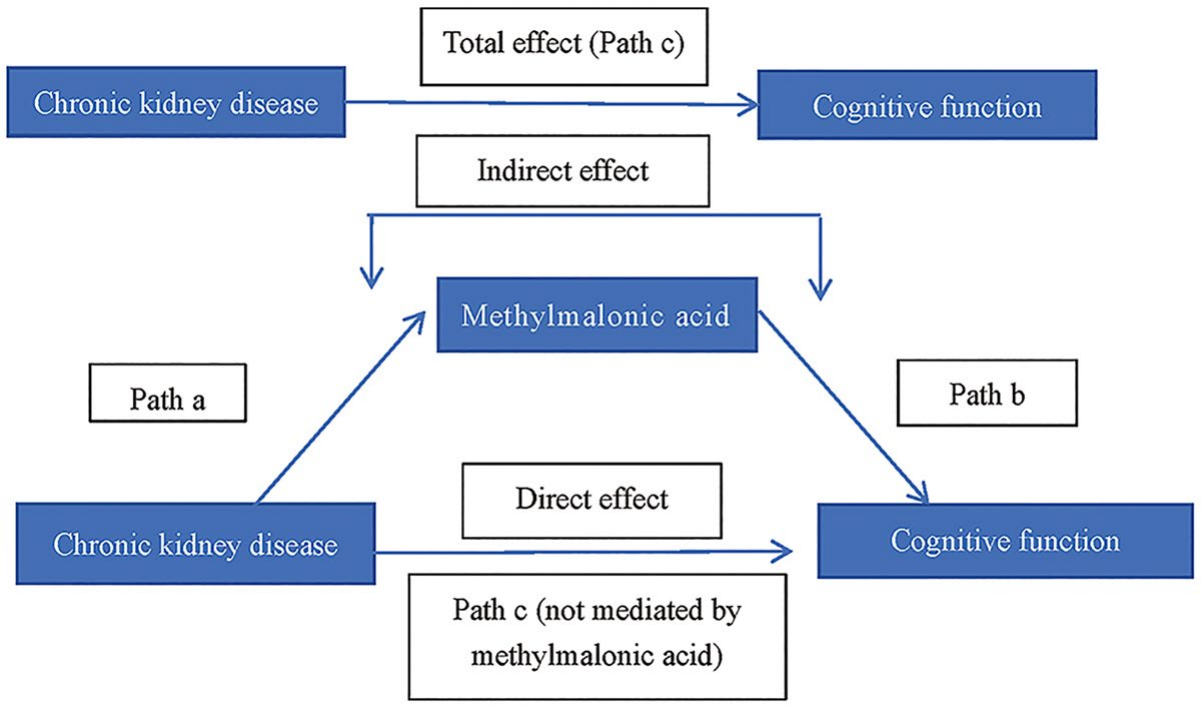
Shows a causal mediation analysis of the relationship between chronic kidney disease and cognitive function, with methylmalonic acid levels as a potential mediator.

To account for the complex sampling framework, all statistical analyses were adjusted for survey design and weighted variables (WTMEC2YR/2). The distribution of continuous variables was assessed using the Shapiro-Wilk test. For normally distributed continuous variables, results were expressed as mean ± standard deviation (SD) and compared using the Student’s t-test. For non-normally distributed continuous variables, data were summarized as median and interquartile range (IQR) and compared using the Mann–Whitney U-test. Categorical variables were presented as frequencies and weighted percentages, with group comparisons conducted using the Chi-squared (χ^2^) test. Linear regression models were developed to evaluate the associations between renal function and cognitive performance, renal function and MMA, and MMA and cognitive performance. The unadjusted model (crude model) included no covariates. Model 1 was adjusted for age, sex, race/ethnicity, educational attainment, marital status, and PIR. Model 2 included the covariates in Model 1 with additional adjustment for BMI, alcohol consumption, smoking status, physical activity, diabetes mellitus, and hypertension. Results were expressed as estimated effect sizes (β) with corresponding standard errors (SE). Restricted cubic spline (RCS) modeling was applied to explore potential nonlinear relationships between eGFR and CF scores, between eGFR and MMA, and between MMA and CF scores. Path analysis was performed to assess the potential mediating role of MMA in the association between renal function and CF (Figure 2). In the first step, the total effect of renal function on CF (path c), adjusted for all covariates, was estimated. Second, the direct influence of renal function on CF after controlling for all covariates and MMA (effect of path c’) was assessed. Third, our study evaluated the indirect impact of CKD on CF mediated through MMA (effects of path a and path b) after all covariates were controlled. Fourth, the proportion of mediation by MMA (i.e. indirect effect/total effect) was appraised (Figure 2). All analyses were finished *via* R 4.4.1. A two-sided p-value < 0.05 denotes statistical significance.

## Results

### Baseline Characteristics of participants

2,437 participants were included in our study. Table 1 presents that among the overall population, 46% were male and 54% were female. Most of them were married (65%), non-Hispanic White (81%), and possessed a college degree or higher (63%), with a median age of 68. Compared with the group having higher cognitive scores, the group with lower cognitive scores was older (75 years old) and had a lower PIR (1.63). Significant differences were observed across the quartiles of cognitive scores in terms of age, sex, race/ethnicity, education, PIR, marital status, diabetes, alcohol consumption, hypertension, occupational physical activity, recreational physical activity, and CF. However, no significant differences were observed in BMI or smoking across cognitive score groups.

**Table 1.** Baseline Characteristics of the study samples.

Characteristic	Overal(2437)	15-71(*n* = 620)	72-88(*n* = 605)	89-106(*n* = 617)	107-170(*n* = 595)	p-value*^3^*
Sex						<0.001
Male	1183 (46%)	339 (47%)	335 (53%)	290 (52%)	219 (38%)	
Female	1254 (54%)	281 (53%)	270 (47%)	327 (48%)	376 (62%)	
Age	68 (63,74)	75 (68,80)	71 (65,78)	69 (64,74)	64 (62,69)	<0.001
Ethnicity						<0.001
Mexican American	206 (3.2%)	77 (7.7%)	46 (3.3%)	47 (2.7%)	36 (1.6%)	
Non-Hispanic White	1224 (81%)	209 (62%)	284 (76%)	333 (83%)	398 (90%)	
Non-Hispanic Black	547 (7.6%)	192 (17%)	152 (10%)	132 (6.9%)	71 (2.8%)	
Other Race	460 (8.1%)	142 (14%)	123 (11%)	105 (7.1%)	90 (5.2%)	
Education level						<0.001
<High school diploma	570 (15%)	319 (41%)	164 (24%)	64 (9.4%)	23 (2.9%)	
High school diploma/ equivalent	579 (22%)	152 (27%)	172 (29%)	158 (25%)	97 (13%)	
>High school diploma	1288 (63%)	149 (31%)	269 (47%)	395 (65%)	475 (84%)	
Marital status						<0.001
Married/cohabitation	1411 (65%)	299 (51%)	352 (61%)	382 (68%)	378 (71%)	
Widow/divorce/separation	891 (31%)	289 (44%)	217 (35%)	204 (28%)	181 (25%)	
Unmarried	135 (4.3%)	32 (5.1%)	36 (4.2%)	31 (3.4%)	36 (4.7%)	
PIR	3.13 (1.70,5.00)	1.63 (1.06,2.72)	2.15 (1.36,4.02)	3.13 (1.89,4.84)	4.76 (2.70,5.00)	<0.001
BMI	28 (25,32)	27 (24,32)	28 (25,32)	29 (25,33)	28 (25,32)	0.383
Creatinine	0.92 (0.77,1.10)	0.98 (0.82,1.23)	0.96 (0.79,1.13)	0.93 (0.78,1.11)	0.86 (0.73,1.04)	<0.001
Sport	700 (0,2,280)	0 (0,960)	360 (0,1,680)	840 (0,2,800)	1,080 (80,2,880)	<0.001
Drinking	1686 (73%)	385 (61%)	414 (68%)	429 (73%)	458 (81%)	<0.001
Smoking						0.042
Never	1198 (49%)	295 (48%)	276 (43%)	303 (47%)	324 (55%)	
Former	924 (39%)	232 (39%)	238 (43%)	239 (41%)	215 (36%)	
Current	315 (11%)	93 (13%)	91 (14%)	75 (12%)	56 (8.8%)	
Diabetes	802 (27%)	263 (38%)	223 (35%)	190 (28%)	126 (16%)	<0.001
BP	1529 (59%)	436 (71%)	386 (64%)	391 (60%)	316 (50%)	<0.001
LBXMMASI	169 (132,229)	191 (143,274)	184 (138,248)	168 (132,229)	159 (125,200)	<0.001
eGFR	72 (61,84)	66 (51,83)	72 (58,83)	73 (61,83)	74 (64,86)	<0.001
CERAD	27 (22,31)	19 (15,22)	23 (20,26)	27 (24,30)	31 (28,34)	<0.001
CERAD_z	0.32 (−0.44,0.94)	−0.90 (−1.52,−0.44)	−0.29 (−0.75,0.17)	0.32 (−0.14,0.78)	0.94 (0.48,1.40)	<0.001
CFDAST	18 (14,22)	12 (9,15)	15 (13,18)	18 (15,20)	22 (19,26)	<0.001
CFDAST_z	0.25 (−0.48,0.98)	−0.85 (−1.40,−0.30)	−0.30 (−0.66,0.25)	0.25 (−0.30,0.62)	0.98 (0.43,1.71)	<0.001
CFDDS	53 (42,64)	28 (22,33)	42 (38,47)	53 (49,57)	67 (62,74)	<0.001
CFDDS_z	0.41 (−0.23,1.04)	−1.04 (−1.38,−0.75)	−0.23 (−0.46,0.06)	0.41 (0.18,0.64)	1.22 (0.93,1.62)	<0.001
cognitive_sum	99 (81,114)	61 (52,67)	82 (76,85)	98 (93,102)	118 (113,127)	<0.001
cognitive_sum_z	0.87 (2.40)	−2.87 (1.24)	−0.71 (0.73)	0.97 (0.75)	3.22 (1.24)	<0.001

**Abbreviations:** BMI: Body Mass Index; PIR: Poverty-to-Income Ratio; eGFR: Estimated Glomerular Filtration Rate; BP: Blood Pressure; CERAD: Consortium to Establish a Registry for AD Word List Learning Test; CERAD_Z: Standardized Score for the Consortium to Establish a Registry for AD Word List Learning Test; CFDAST: Animal Fluency Test; CFDAST_z: Standardized Score for Animal Fluency Test; CFDDS: Digit Symbol Substitution Score; CFDDS_z: Standardized Score for Digit Symbol Substitution.

### Relationship between renal function and CF

Table 2 presents the statistically significant association between GFR and cognitive scores. In the unadjusted model, each one-unit increase in GFR was associated with an average increase of 0.018 points in cognitive score. In Model 2, a marked positive association was observed between GFR and cognitive scores (β = 0.007, 95% CI: 0.003–0.010, *p* = 0.001), indicating that each additional unit of GFR corresponded to an increase of 0.007 points in cognitive score.

**Table 2.** Regression analysis exploring the relationship between renal function and CF.

	Standardized β	β	95% CI	p-value
Crude Model	0.15070	0.018	0.013, 0.024	<0.001
Model 1	0.0611175	0.007	0.004, 0.011	<0.001
Model 2	0.05511	0.007	0.003, 0.010	0.001

Crude model: Non-adjusted.

Model 1: Adjusted for age, sex, ethnicity, education level, marital status, PIR.

Model 2: Adjusted for age, sex, ethnicity, education level, marital status, PIR, BMI, drinking, smoking, sport, diabetes, BP.

**Figure 3.**
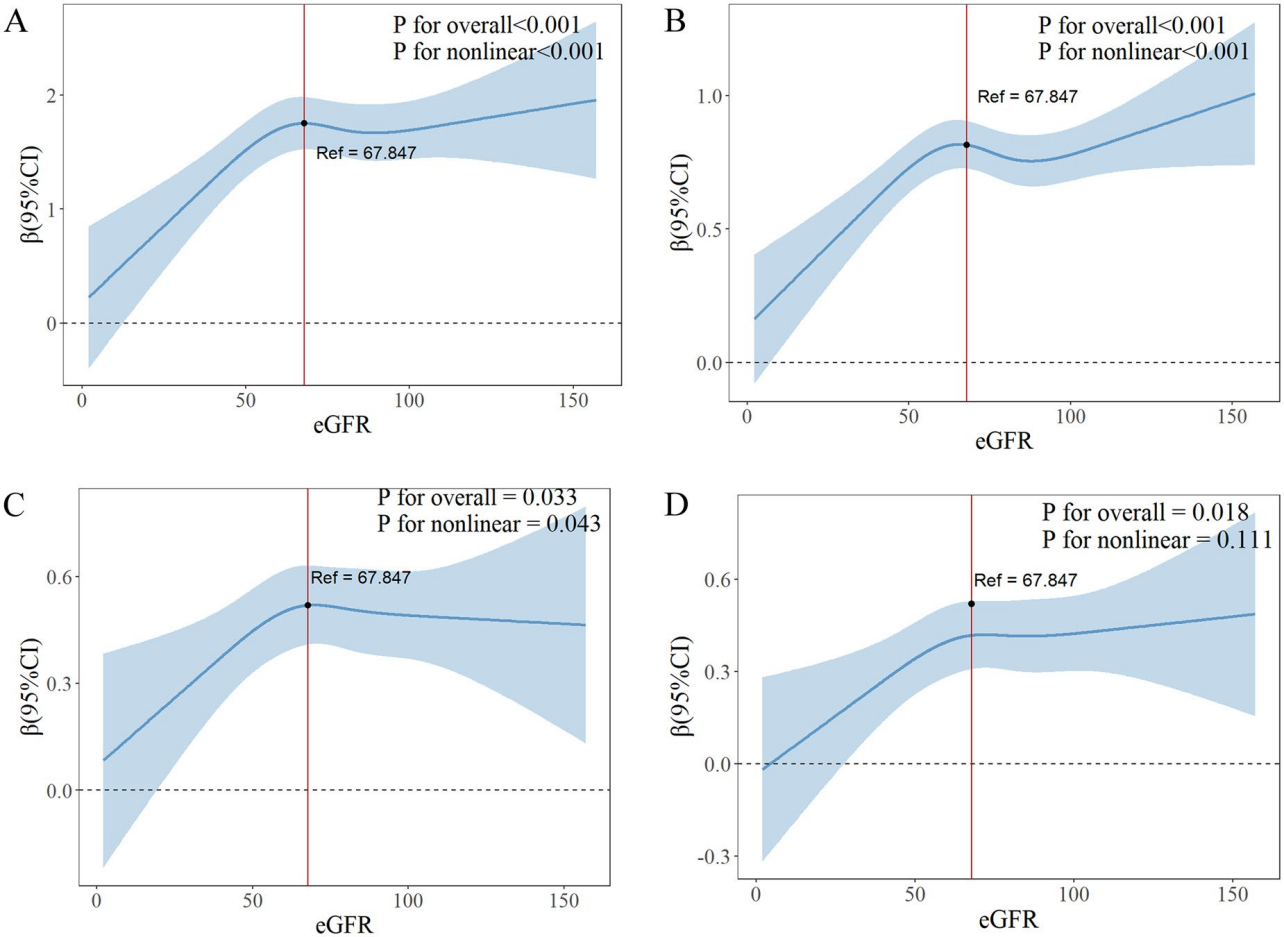
Relationship between chronic kidney disease and cognition. (A) cognitive sum_z (The blue shaded area represents the confidence interval of the estimate); (B) CFDDS_z (The blue shaded area represents the confidence interval of the estimate); (C) CERAD_z (The blue shaded area represents the confidence interval of the estimate); (D) CFDAST_z (The blue shaded area represents the confidence interval of the estimate).

Tables S1-S3 in Supplementary File 1 detail the associations of GFR with individual CF measures (CFDAST_z, CFDDS_z, and CERAD_Z). After adjustment for all selected covariates, a significant association was found between GFR and CFDDS_z (β = 0.003, 95% CI: 0.001–0.006, *p* = 0.015), whereas no significant associations were observed between GFR and either CFDAST_z or CERAD_Z (*p* > 0.05). RCS analyses were conducted to assess potential nonlinear associations between eGFR and total CF score, CFDDS score, CFDAST score, and CERAD score. The results demonstrated significant nonlinear associations between eGFR and total CF score (p for overall < 0.001; p for nonlinearity < 0.001; Figure 3 (A)), CFDDS score (p for overall < 0.001; p for nonlinearity < 0.001; Figure 3(B)), and CERAD score (p for overall = 0.033; p for nonlinearity = 0.043; Figure 3 (C)). A linear association was observed between eGFR and CFDAST score (p for overall = 0.018; p for nonlinearity = 0.111; Figure 3(D)).

### Relationship between renal function and MMA

**Figure 4.**
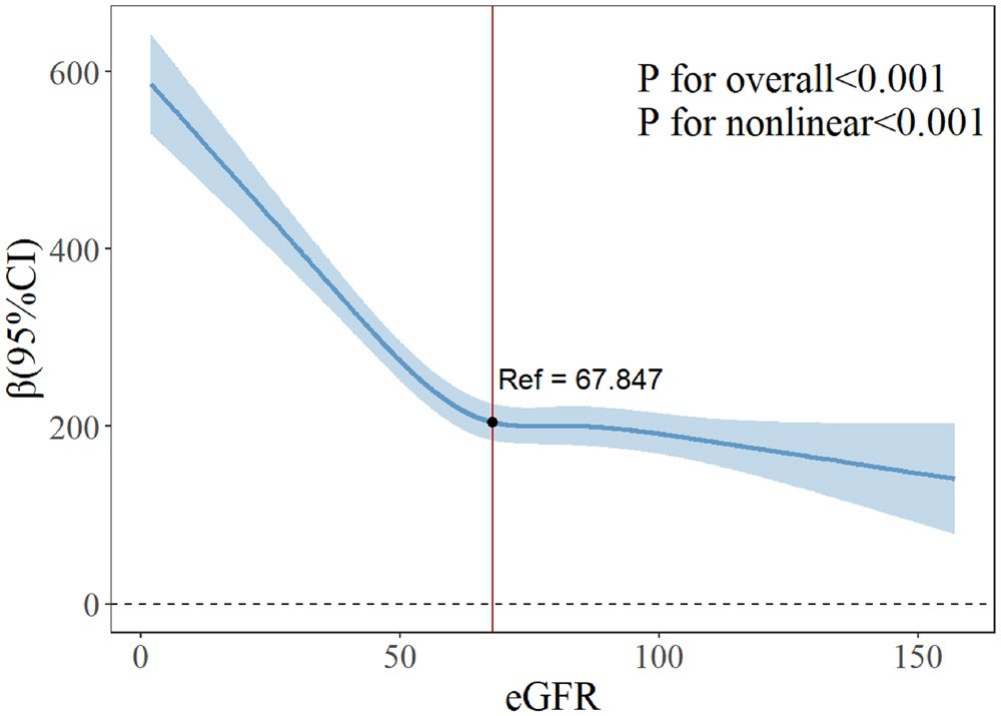
Correlation between chronic kidney disease and methylmalonic acid (The blue shaded area represents the confidence interval of the estimate).

Table 3 summarizes regression analyses indicating a marked association between GFR and MMA levels. In the unadjusted model, GFR was inversely associated with MMA levels. In Model 1, each unit increase in GFR corresponded to a reduction of 2.148 units in MMA levels. After adjustment for all confounding variables, the inverse association persisted. RCS analysis further revealed a significant nonlinear association between eGFR and MMA (p for overall < 0.001; p for nonlinearity < 0.001; Figure 4).

**Table 3.** Regression analysis examining the association between renal function and MMA.

	Standardized β	β	95% CI	p-value
Crude Model	−0.2711	−2.148	−2.570, −1.727	<0.001
Model 1	−0.24188	−1.923	−2.412, −1.434	<0.001
Model 2	−0.237008	−1.886	−2.386, −1.386	<0.001

Crude model: Non-adjusted.

Model 1: Adjusted for age, sex, ethnicity, education level, marital status, PIR.

Model 2: Adjusted for age, sex, ethnicity, education level, marital status, PIR, BMI, drinking, smoking, sport, diabetes, BP.

### Relationship between MMA and CF

Table 4 demonstrates a significant negative association between MMA levels and cognitive performance.

**Table 4.** Regression analysis investigating the relationship between MMA and CF.

	Standardized β	β	95% CI	p-value
Crude Model	−0.149574	−0.00234	−0.00331, −0.00137	<0.001
Model 1	−0.0615827	−0.00096	−0.00160, 0.00031	0.006
Model 2	−0.0558818	−0.00087	−0.00154, 0.00020	0.015

Crude model: Non-adjusted.

Model 1: Adjusted for age, sex, ethnicity, education level, marital status, PIR.

Model 2: Adjusted for age, sex, ethnicity, education level, marital status, PIR, BMI, drinking, smoking, sport, BP, diabetes.

**Figure 5.**
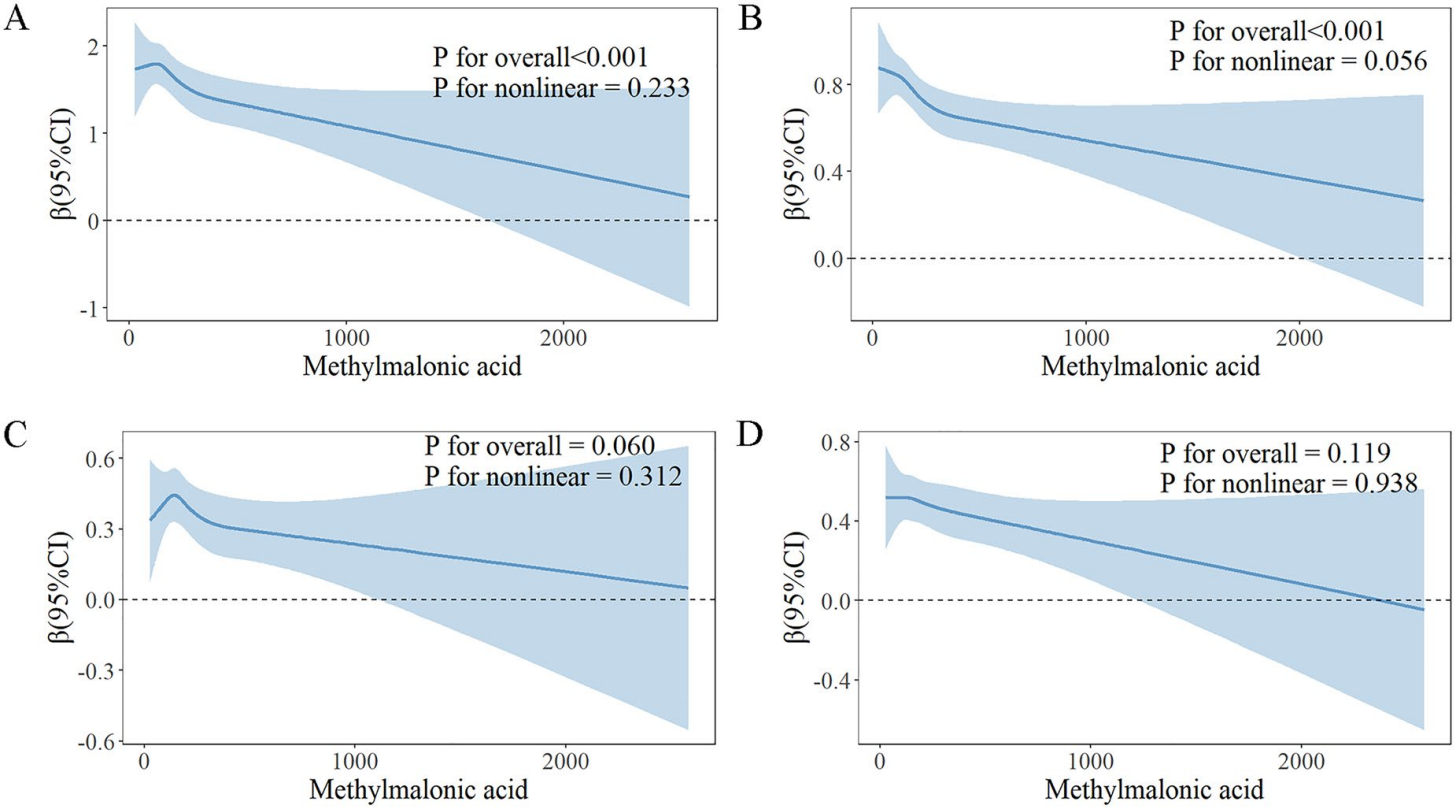
Association of methylmalonic acid with cognition. (A) cognitive sum_z (The blue shaded area represents the confidence interval of the estimate); (B) CFDDS_z (The blue shaded area represents the confidence interval of the estimate); (C) CFDAST_z (The blue shaded area represents the confidence interval of the estimate); (D) CERAD_z (The blue shaded area represents the confidence interval of the estimate).

Tables S4-S6 in Supplementary File 2 present the associations between MMA levels and individual CF scores (CFDAST_z, CFDDS_z, and CERAD_Z). Following adjustment for all covariates, a significant association was found between MMA levels and CFDDS_z (β= −0.00040, 95% CI: −0.00070 to −0.00011, *p* = 0.011), whereas no significant associations were detected between MMA levels and CFDAST_z or CERAD_Z (*p* > 0.05). RCS analyses indicated that MMA was linearly associated with total CF score (p for overall < 0.001; p for nonlinearity = 0.233; Figure 5 (A)) and CFDDS score (p for overall < 0.001; p for nonlinearity = 0.056; Figure 5(B)), but not significantly associated with CFDAST score (p for overall = 0.060; p for nonlinearity = 0.312; Figure 5(C)) or CERAD score (p for overall = 0.119; p for nonlinearity = 0.938; Figure 5(D)).

### The mediating role of MMA in the relationship between renal function and CF

**Figure 6.**
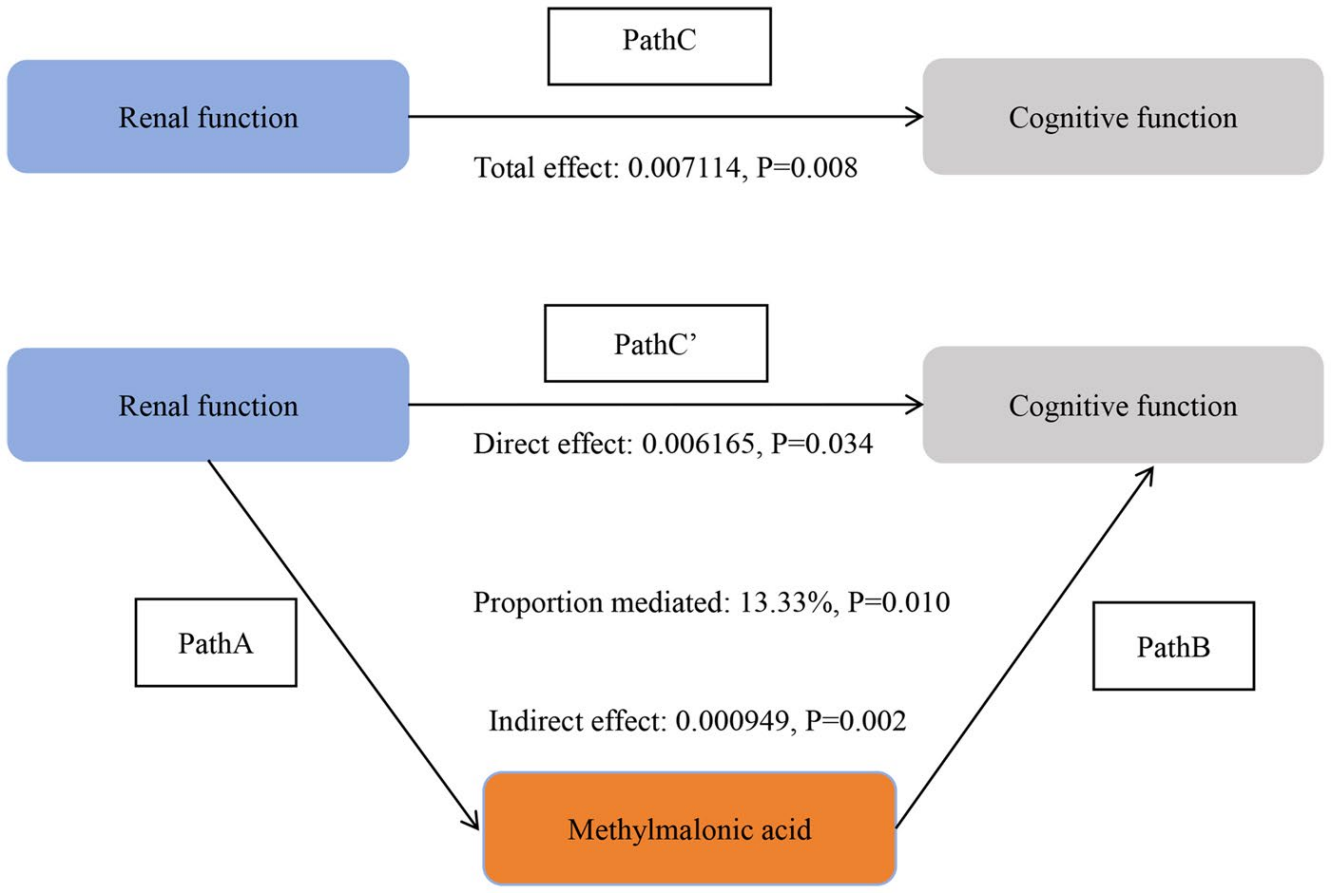
Direct and Indirect Effects of renal function on CF Mediated by MMA. In the schematic diagram of mediation analysis, Path C represents the total effect, while Path C′ denotes the direct effect. The indirect effect is estimated as the product of Path A and Path B (Path A*B). The mediation proportion is calculated as [indirect effect/(indirect effect + direct effect)] × 100%. Adjusted Analyses: Adjusted for age, sex, ethnicity, education level, marital status, PIR, drinking, smoking, BMI, sport, BP, diabetes.

The potential mediating effect of MMA in the association between CKD and CF was further evaluated. Table 5 and Figure 6 shows that MMA significantly mediated the association between GFR and cognitive scores (proportion mediated: 13.33%; indirect effect: β = 0.000949, 95% CI: 0.000419–0.002463, *p* = 0.002) after adjusting for covariates. The direct association between GFR and cognitive scores remained significant in the absence of MMA mediation (β = 0.006165, 95% CI: 0.000271–0.010456, *p* = 0.034).

**Table 5.** Direct and indirect effects of renal function on CF mediated by MMA.

	Unadjusted Analyses				Adjusted Analyses			
	Estimate	95%CI lower	95%CI upper	p-value	Estimate	95%CI lower	95%CI upper	p-value
Indirect effect	0.004008	0.002292	0.005788	<0.001	0.000949	0.133384	0.002463	0.002
Direct effect	0.015163	0.008531	0.020444	<0.001	0.006165	0.000271	0.010456	0.034
Total effect	0.019171	0.012607	0.024265	<0.001	0.007114	0.000271	0.011855	0.008
Proportion mediated	0.209066	0.123157	0.357518	<0.001	0.133384	0.051474	0.781719	0.010

Unadjusted Analyses: Non-adjusted.

Adjusted Analyses: Adjusted for age, sex, ethnicity, education level, marital status, PIR, drinking, smoking, BMI, sports, BP, and diabetes.

**Figure 7.**
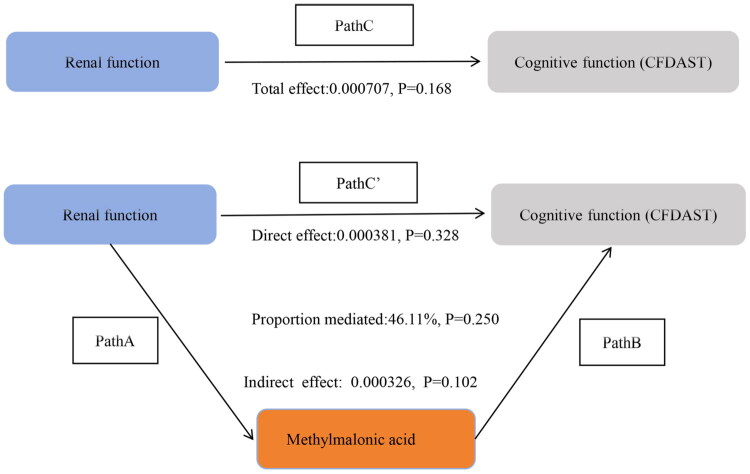
Direct and indirect effects of methylmalonic acid-mediated chronic kidney disease on cognitive function (CFDAST). In the schematic diagram of mediation analysis, Path C represents the total effect, while Path C′ denotes the direct effect. The indirect effect is estimated as the product of Path A and Path B (Path A*B). The mediation proportion is calculated as [indirect effect/(indirect effect + direct effect)] × 100%. Adjusted Analyses: Adjusted for age, sex, ethnicity, education level, marital status, PIR, drinking, smoking, BMI, sport, BP, diabetes.

**Figure 8.**
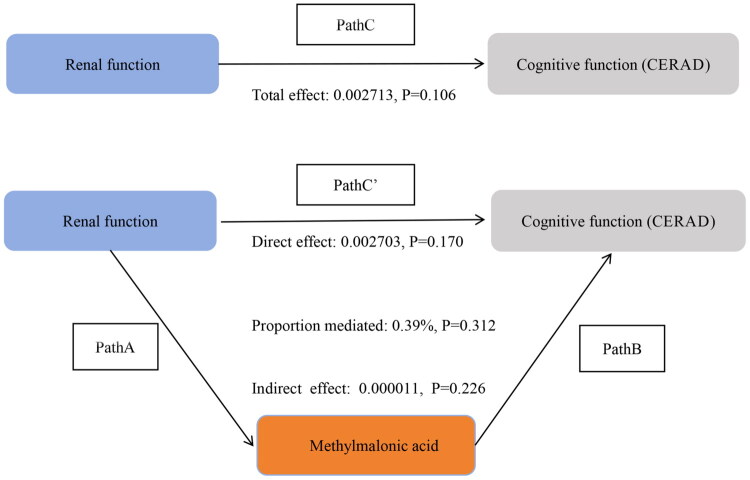
Direct and indirect effects of methylmalonic acid-mediated chronic kidney disease on cognitive function (CERAD). In the schematic diagram of mediation analysis, Path C represents the total effect, while Path C′ denotes the direct effect. The indirect effect is estimated as the product of Path A and Path B (Path A*B). The mediation proportion is calculated as [indirect effect/(indirect effect + direct effect)] × 100%. Adjusted Analyses: Adjusted for age, sex, ethnicity, education level, marital status, PIR, drinking, smoking, BMI, sport, BP, diabetes.

**Figure 9.**
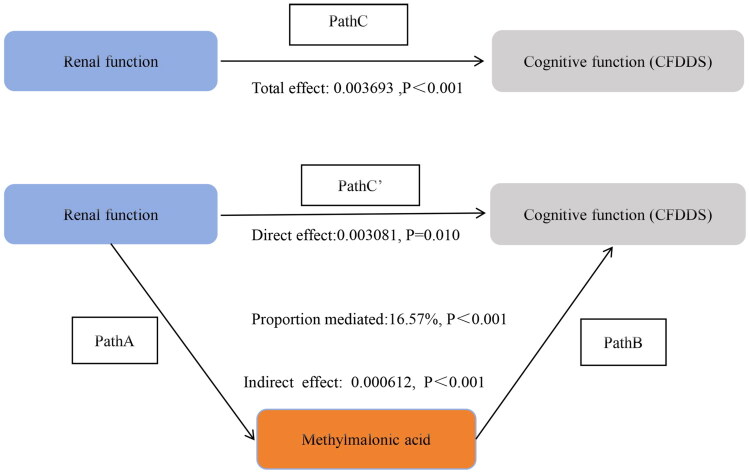
Direct and indirect effects of methylmalonic acid-mediated chronic kidney disease on cognitive function (CFDDS). In the schematic diagram of mediation analysis, Path C represents the total effect, while Path C′ denotes the direct effect. The indirect effect is estimated as the product of Path A and Path B (Path A*B). The mediation proportion is calculated as [indirect effect/(indirect effect + direct effect)] × 100%. Adjusted Analyses: Adjusted for age, sex, ethnicity, education level, marital status, PIR, drinking, smoking, BMI, sport, BP, diabetes.

Tables S7-S9 in Supplementary File 3 and Figures 7–9 indicate that MMA levels did not significantly mediate the associations between GFR and CFDAST_z or CERAD_Z. However, MMA showed a notable mediating effect in the association between GFR and CFDDS_z (proportion mediated: 16.57%; indirect effect: β = 0.000612, 95% CI: 0.000205–0.001094, *p* < 0.001). The proportion of mediation by MMA in the GFR-CFDDS_z association was lower than that observed for the GFR-overall cognitive score association.

### Sensitivity analysis

Given the potential impact of vitamin B12 status, inflammatory markers, and depressive symptoms on the association between CF and renal function, our study included serum vitamin B12 concentration, white blood cell count, lymphocyte percentage, and depressive symptoms as additional covariates in a sensitivity analysis. As shown in Supplementary File 4, after adjusting for these covariates, renal function remained significantly associated with the total cognitive score (β = 0.006, 95% CI: 0.002–0.010, *p* = 0.008) and with the CFDDS score (β = 0.003, 95% CI: 0.000–0.005, *p* = 0.024). No significant associations were found between renal function and either the CFDAST or CERAD scores. Renal function was significantly associated with MMA levels (β=-1.882, 95% CI: −2.382 to −1.381, *p* < 0.001). No significant associations were observed between MMA and the total cognitive score, CFDDS, CFDAST, or CERAD scores.

## Discussion

This study sheds light on the association between renal function and CF in older adults, with particular emphasis on the mediating role of MD. A significant association was observed between impaired renal function and cognitive impairment, whereas biomarkers of MD, such as elevated MMA, were inversely associated with cognitive performance. MD in patients with CKD may constitute one of the underlying mechanisms contributing to cognitive impairment. Accordingly, dementia screening and longitudinal monitoring in patients with CKD carry considerable clinical relevance. In the early stages of CKD, dynamic assessment of CF through measurement of serum MMA concentrations may facilitate timely detection and intervention for cognitive impairment.

Our findings demonstrated a marked inverse association between eGFR and CF. A substantial proportion of patients with CKD exhibit cognitive impairment, and CKD is among the strongest risk factors for both mild cognitive impairment (MCI) and dementia [8]. However, the precise link between CKD and cognitive impairment or its intermediate phenotypes remains incompletely understood. The underlying pathophysiology of CKD-associated cognitive impairment is multifactorial, involving genetic predisposition, uremic toxin accumulation, vascular dysfunction, and neuroinflammation [7]. The prevalence of atherosclerosis is high in the CKD population, and endothelial dysfunction is nearly ubiquitous [27]. Due to impairment of both glomerular and tubular function, the failing kidney retains uremic toxins, including guanidino compounds, indoxyl sulfate, homocysteine, trimethylamine N-oxide, kynurenine, and quinolinic acid. In uremia, the albumin-binding capacity is compromised, increasing the fraction of free (unbound) toxins. These free toxins are more readily able to cross the BBB. Moreover, indoxyl sulfate disrupts BBB integrity *via* activation of the aryl hydrocarbon receptor [28]. Once within the brain parenchyma, uremic toxins are poorly cleared by the glymphatic system, leading to their accumulation. These toxins damage neural progenitor cells, the vascular system, and monoaminergic neurons, thereby contributing substantially to cognitive impairment in CKD [29]. CKD patients also exhibit impaired endothelium-dependent microvascular reactivity [30]; endothelial dysfunction disrupts microcirculatory perfusion and compromises BBB integrity, further predisposing to cognitive impairment.

In end-stage kidney disease (ESKD), renal replacement therapies, such as peritoneal dialysis, hemodialysis, and kidney transplantation, are commonly required.

Among individuals receiving hemodialysis, the prevalence of cognitive impairment is as high as 60% [31]. Hemodynamic fluctuations during hemodialysis may result in inadequate cerebral perfusion, thereby exacerbating cognitive dysfunction. Several studies have reported that patients undergoing peritoneal dialysis demonstrate better cognitive performance than those on hemodialysis [32,33]. Peritoneal dialysis, by providing continuous clearance of middle-molecular-weight toxins, appears to offer greater neurocognitive protection. Following kidney transplantation, cognitive impairment and structural brain abnormalities appear at least partially reversible [34].

After transplantation, renal function is restored, toxin clearance is markedly improved, cerebral hemodynamics normalize, and both inflammation and OS are substantially reduced, all of which contribute to the improvement of CF. Mitochondria, recognized as the cellular “powerhouses,” are responsible for ATP production and participate in numerous intracellular metabolic pathways. MD not only disrupts cellular energy metabolism but also triggers OS, inflammatory responses, and apoptosis, thereby impairing neuronal function and survival. MMA, a metabolic byproduct intimately linked to mitochondrial metabolism, is elevated in the context of MD and serves as a biomarker indicative of mitochondrial impairment [35]. MMA is produced during the breakdown of fatty acids and amino acids and is normally converted to succinyl-CoA, entering the TCA cycle [14]. Abnormal MMA accumulation has been associated with MD and OS. Declining renal function reduces the body’s ability to eliminate toxins, with MMA being one of the most notable accumulating metabolites. Studies have demonstrated a significant inverse association between serum MMA levels and residual renal function. As renal function declines, MMA accumulates progressively, potentially exerting deleterious effects on cerebral function [22]. MMA has been shown to inhibit the mitochondrial respiratory chain, impair energy metabolism in the hippocampus, and induce lipid peroxidation in the cerebral cortex, thereby increasing OS and causing neuronal injury. Furthermore, MMA may disrupt neurotransmitter homeostasis and impair neural signaling pathways, leading to adverse effects on CF [36]. Multiple investigations have confirmed a significant inverse association between serum MMA concentrations and CF. Excessive accumulation of MMA results primarily from a deficiency in the active coenzyme form of vitamin B12 (VitB12) or from inactivation of mitochondrial methylmalonyl-CoA mutase (MUT) [37]. Excessive MMA accumulation disrupts the electron transport chain, further impairing mitochondrial energy metabolism and generating reactive oxygen species (ROS) [18,38]. Furthermore, growing evidence indicates that MMA accumulation modulates the mitochondrial respiratory chain, stimulating ROS release and exacerbating MD and OS [18,19].

MD and OS are pivotal in the pathogenesis of CKD. Mitochondria are the primary energy producers in renal cells but are also particularly susceptible to OS-induced damage. CKD patients exhibit mitochondrial structural and functional alterations in renal tissues, including morphological remodeling, increased OS, and decreased mitochondrial biogenesis and ATP production [39]. MD contributes to decreased mitochondrial respiratory capacity, elevated ROS production, and mitochondrial DNA damage. Despite its importance, the impact of MD on the initiation and progression of CKD remains underexplored. Considering its central role in renal pathology, strategies aimed at preserving mitochondrial integrity and preventing dysfunction may offer therapeutic potential for the management of various renal diseases.

Mitochondria possess a robust antioxidant system comprising enzymatic components like superoxide dismutase 2 (SOD2), glutathione peroxidase (GPX), catalase, thioredoxin reductase, low-molecular-weight molecules like reduced glutathione, ascorbic acid, coenzyme Q, and vitamin E, which collectively serve as scavengers of ROS [40]. Dysfunction of these antioxidant defenses results in intracellular ROS accumulation, initiating pathophysiological cascades of cellular injury and apoptosis, culminating in OS and subsequent cell death. Under both extracellular and intracellular stress conditions, mitochondrial damage may provoke a range of abnormalities, including perturbations in energy metabolism, OS, calcium homeostasis, mitochondrial dynamics and biogenesis, synaptic dysfunction, neuronal loss, and cognitive deficits [41]. The brain is particularly vulnerable to OS [42]. OS-induced brain injury can adversely impact central nervous system (CNS) function, reduce long-term potentiation, and disrupt synaptic signaling and brain plasticity [43,44]. Excessive ROS production exceeding antioxidant capacity leads to widespread protein oxidation and lipid peroxidation, resulting in oxidative damage, cellular degeneration, and progressive cognitive and functional decline [45]. Hippocampus, amygdala, and cerebellar granule cells are especially susceptible to OS, thereby undergoing functional impairment at early stages [46]. Consequently, OS-induced damage to these brain regions results in severe cognitive, behavioral, and functional deficits. The accumulation of MMA in body fluids has been shown to elevate cortical levels of interleukin-1β (IL-1β) and tumor necrosis factor-alpha (TNF-α) and upregulate pro-inflammatory markers like inducible nitric oxide synthase (iNOS) and 3-nitrotyrosine (3-NT), ultimately promoting the progression of cognitive dysfunction [25]. The buildup of MMA in cerebrospinal fluid may exert neurotoxic effects [47]. Elevated MMA levels inhibit the respiratory chain, impairing energy metabolism in hippocampal tissues [48]. Moreover, neurological deficits observed in methylmalonic acidemia are attributed to MMA-induced cortical lipid peroxidation [49]. Our findings indicate a significant inverse association between GFR and MMA levels, and a positive association between MMA concentrations and cognitive dysfunction. Additionally, GFR shows a significant positive association with cognitive performance scores. Accordingly, it is hypothesized that renal function is significantly associated with CF in elderly individuals, with methylmalonic acidemia potentially mediating this relationship. Although renal impairment is a recognized contributor to CF decline, cognitive impairment may reciprocally impact renal function *via* multiple pathways. For example, cognitive deficits can impair self-care capabilities, reducing adherence to medical regimens for chronic conditions such as CKD, thus accelerating renal disease progression [50]. Moreover, cognitive impairment can adversely affect emotional well-being, contributing to conditions such as anxiety and depression, which in turn may influence renal function through multiple biological and behavioral pathways [50,51]. Furthermore, renal and cognitive impairments may share common underlying pathophysiological mechanisms. OS and systemic inflammation are central to the pathogenesis of both CKD and cognitive impairment. OS induces cellular damage and dysfunction, while chronic inflammation compromises vascular integrity, impairing perfusion to the kidneys and brain alike. Vascular injury can further disrupt hemodynamics, fostering parallel deterioration of renal and CF. Future studies are warranted to elucidate these complex causal interrelationships and deepen understanding of the bidirectional association between kidney function and cognition [16,52].

In the present study, no significant differences in BMI or smoking were observed across groups stratified by cognitive performance. Although these findings may seem inconsistent with prior evidence linking obesity and smoking to cognitive impairment, several plausible explanations exist. BMI, as a cardiovascular risk factor, is strongly associated with metabolic syndrome, diabetes, and cardiovascular disease, conditions known to impair cognition. However, the relationship between BMI and CF is likely nonlinear and influenced by multiple confounders, some exerting more direct cognitive effects [53]. Notably, our cohort mainly comprised well-educated, non-Hispanic white individuals with healthier lifestyles and heightened health awareness, potentially mitigating the adverse impacts of elevated BMI on cognition. Smoking, another established cardiovascular risk factor, mechanistically relates to OS, systemic inflammation, and vascular injury, all contributing to cognitive deficits. Nevertheless, the effect of smoking on CF may vary markedly between individuals. In our sample, the low prevalence of smokers (11%) combined with overall favorable health status may have limited the detectable influence of smoking on cognition. Additionally, smokers in this cohort might have engaged in compensatory health behaviors such as improved diet or regular physical activity, partially offsetting smoking-associated cognitive risks. It is also important to acknowledge that the cross-sectional design limits causal inference and temporal resolution. The high educational attainment and good health profile of participants may further attenuate observable associations between BMI, smoking, and CF. Future longitudinal studies encompassing more diverse populations are necessary to more fully assess the impact of BMI and smoking on cognitive outcomes.

In this study, serum MMA levels significantly mediated the association between renal function and cognitive performance, accounting for 13.33% of the total effect. Although this proportion is relatively modest, it carries significant clinical implications. Specifically, elevated MMA levels may indicate MD, a recognized mechanism contributing to cognitive impairment. Therefore, monitoring serum MMA concentrations could facilitate the early identification of patients at risk for cognitive impairment secondary to renal dysfunction. From a clinical standpoint, early screening and intervention are essential. For patients with CKD, particularly those experiencing declining renal function, routine cognitive assessments should be incorporated into clinical practice. Brief cognitive screening instruments, such as the Animal Fluency Test (AFT) or DSST, may serve as practical initial tools. Upon detection of cognitive impairment, timely intervention should be initiated to mitigate or slow cognitive impairment. Furthermore, serum MMA may serve as a potential biomarker for assessing cognitive impairment risk in CKD patients. Regular monitoring of MMA levels may enable the early identification of high-risk individuals. In those with elevated MMA, vitamin B12 supplementation or other nutritional interventions may be considered to reduce MMA concentrations and potentially improve cognitive outcomes.

The current study has several strengths. It represents the first population-based investigation elucidating the mediating role of MD in the association between CKD and CF. Importantly, these observed associations remained robust after adjusting for potential confounders, including sociodemographic characteristics, lifestyle factors, and comorbid health conditions. Additionally, four cognitive tests, CERAD Immediate Recall (CERAD-IR), CERAD Delayed Recall (CERAD-DR), DSST, and AFT, were employed to provide a comprehensive evaluation of cognitive performance across multiple domains.

Nevertheless, our study also has certain limitations. First, the CF indices used in this study (AFT, CERAD, and DSST scores) and data processing methods utilized for generating composite scores for overall cognitive performance may lead to potential bias in our results. Second, the cross-sectional design renders it impossible to establish temporal or causal correlations. Long-term follow-up interventional studies are warranted for better understanding renal function and cognitive impairment and verifying the impact of MD on the biological mechanisms.

While this study elucidated the mediating role of MMA in the relationship between CKD and cognitive impairment, the exclusive use of MMA as a biomarker for MD has limitations. MD is a complex pathophysiological process involving multiple metabolic pathways and diverse biomarkers. Beyond MMA, lactate, pyruvate, and the lactate-to-pyruvate ratio (LPR) are established indicators of MD. Lactate and pyruvate are key metabolites in glycolysis and oxidative phosphorylation. Under normal physiological conditions, the LPR remains stable; however, mitochondrial impairment results in increased lactate production and elevated LPR, serving as a sensitive indicator of mitochondrial impairment[54]. Moreover, elevated lactate levels have also been associated with cognitive impairment. Previous studies suggest that increased lactate may impair neuronal function and cognitive performance through the induction of OS and inflammatory responses [55]. Nevertheless, this study did not include lactate, pyruvate, or LPR as biomarkers of MD, which may limit the comprehensiveness of our understanding regarding the role of MD in the association between CKD and cognitive impairment. Future research should consider the simultaneous assessment of multiple biomarkers of MD to more thoroughly evaluate their contributions to CKD-related cognitive impairment.

In the present study, certain cognitive domains demonstrated significant associations with kidney function or MMA levels, while others did not. This discrepancy may arise from several factors. First, the sensitivity of cognitive assessments varies across domains; for example, the AFT primarily measures language and verbal fluency, whereas the DSST evaluates executive function and processing speed. Some tests may be more responsive to changes in renal function or MMA, while others may not detect such alterations effectively. Moreover, distinct cognitive domains depend on different brain regions and neural networks; memory relies heavily on the hippocampus and medial temporal lobes, while executive function involves the prefrontal cortex. Consequently, impaired renal function or elevated MMA may disproportionately affect specific brain areas. Participant characteristics such as age, education level, and general health status may also influence cognitive test performance. Individuals with higher education may perform better, potentially obscuring the effects of kidney dysfunction or elevated MMA. Furthermore, kidney dysfunction and elevated MMA may impair cognition *via* divergent pathophysiological pathways. Elevated MMA may predominantly disrupt mitochondrial function and energy metabolism, affecting executive function and processing speed more than memory. Both conditions may influence cognition through mediators such as nutritional deficits, inflammation, and OS. Different cognitive domains may vary in susceptibility to these mediators. Cognitive impairment manifests variably as memory loss, decreased attention, slowed processing, and executive dysfunction, with differential sensitivity to renal impairment. Notably, CF in dialysis patients fluctuates, often declining during sessions and improving before or shortly after, which may introduce measurement bias [36].

## Conclusion

This cross-sectional study identified a potential association between CKD and CF in an elderly U.S. population. MD, assessed by MMA levels, may partially mediate the relationship between renal function and cognitive performance, particularly showing significant associations with eGFR and MMA within DSST subdomains. Therefore, MMA may represent a promising biomarker for cognitive impairment risk in CKD patients.

## Supplementary Material

Supplementary File 3 R2.docx

Supplementary File 1.docx

Supplementary File 2 R2.docx

Supplementary FIle 4 R2.docx

## Data Availability

The datasets analyzed during the current study are available in the National Center for Health Statistics (NCHS), https://www.cdc.gov/nchs/nhanes/about/index.html.
