# Supplementary material for: Renal function and cognitive performance in older adults: a NHANES-based mediation analysis of methylmalonic acid as a marker of mitochondrial dysfunction
Source: Ren Fail. 2025 Nov 17;47(1):2577843. doi: 10.1080/0886022X.2025.2577843 (PMC12624903; doi:10.1080/0886022X.2025.2577843)
Supplement: Supplementary File 3 R2.docx [file IRNF_A_2577843_SM1670.docx]

**Supplementary File 3** Direct and indirect effects of renal function on cognitive function mediated by methylmalonic acid

**Table S7** Association between chronic kidney disease and cognition (CFDAST_z ~ eGFR)

Standardized β

**β **95% CI***^1^* **p-value****

Crude Model 0.097 0.005 0.002, 0.008 <0.001

Model1 0.037 0.002 0.000, 0.005 0.105

Model2 0.029 0.002 -0.001, 0.004 0.171

Crude model: Non-adjusted.

Model 1: Adjusted for age, gender, ethnicity, education level, marital status,PIR,

Model 2: Adjusted for age, gender, ethnicity, education level, marital status, PIR,BMI,Drink,Smoking,Sport,Diabetes,BP

**Table S8** Association between chronic kidney disease and cognition(CFDDS_z ~ eGFR)

Standardized β **β **95% CI***^1^* **p-value****

Crude Model 0.148 0.007 0.005, 0.010 <0.001

Model1 0.069 0.003 0.001, 0.006 0.011

Model2 0.067 0.003 0.001, 0.006 0.015

Crude model: Non-adjusted.

Model 1: Adjusted for age, gender, ethnicity, education level, marital status,PIR,

Model 2: Adjusted for age, gender, ethnicity, education level, marital status, PIR,BMI,Drink,Smoking,Sport,Diabetes,BP

**Table S9** Association between chronic kidney disease and cognition（CERAD_z~ eGFR）

Standardized β **β **95% CI***^1^* **p-value****

Crude Model 0.123 0.006 0.004, 0.009 <0.001

Model1 0.044 0.002 0.000, 0.004 0.044

Model2 0.039 0.002 0.000, 0.004 0.069

Crude model: Non-adjusted.

Model 1: Adjusted for age, gender, ethnicity, education level, marital status,PIR,

Model 2: Adjusted for age, gender, ethnicity, education level, marital status, PIR,BMI,Drink,Smoking,Sport,Diabetes,BP
