# Supplementary material for: Renal function and cognitive performance in older adults: a NHANES-based mediation analysis of methylmalonic acid as a marker of mitochondrial dysfunction
Source: Ren Fail. 2025 Nov 17;47(1):2577843. doi: 10.1080/0886022X.2025.2577843 (PMC12624903; doi:10.1080/0886022X.2025.2577843)
Supplement: Supplementary File 1.docx [file IRNF_A_2577843_SM1669.docx]

**Supplementary File 1** Exploring the association between renal function and cognition

**Table S1** Direct and indirect effects of methylmalonic acid-mediated renal function on cognitive function (CFDAST_z)

Unadjusted Analyses Adjusted Analyses

Estimate 95%CI lower 95%CI upper P-value Estimate 95%CI lower 95%CI upper P-value

Indirect effect 0.001346 0.000519 0.001947 ＜0.001 0.000326 -0.000058 0.001006 0.102000

Direct effect 0.003366 0.001400 0.01400 ＜0.001 0.000381 -0.001221 0.003605 0.328000

Total effect 0.004712 0.002685 0.007656 ＜0.001 0.000707 -0.000758 0.003935 0.168000

Proportion mediated 0.285655 0.089060 0.505990 ＜0.001 0.461120 -1.827735 3.175432 0.250000

Unadjusted Analyses: Non-adjusted.

Adjusted Analyses: Adjusted for age, gender, ethnicity, education level, marital status,PIR, Drink, smoking, BMI, Sport,BP,Diabetes.

**Table S2** Direct and indirect effects of methylmalonic acid-mediated renal function on cognitive function (CFDDS_z)

Unadjusted Analyses Adjusted Analyses

Estimate 95%CI lower 95%CI upper P-value Estimate 95%CI lower 95%CI upper P-value

Indirect effect 0.001815 0.000979 0.002410 0.000000 0.000612 0.000205 0.001094 0.000000

Direct effect 0.006005 0.003081 0.007669 0.000000 0.003081 0.000699 0.004497 0.010000

Total effect 0.007820 0.004847 0.009415 0.000000 0.003693 0.001379 0.005080 0.000000

Proportion mediated 0.232064 0.130480 0.399668 0.000000 0.165709 0.059715 0.521986 0.000000

Unadjusted Analyses: Non-adjusted.

Adjusted Analyses: Adjusted for age, gender, ethnicity, education level, marital status,PIR, Drink, smoking, BMI, Sport,BP,Diabetes.

**Table S3** Direct and indirect effects of methylmalonic acid-mediated renal function on cognitive function (CERAD_z)

Unadjusted Analyses Adjusted Analyses

Estimate 95%CI lower 95%CI upper P-value Estimate 95%CI lower 95%CI upper P-value

Indirect effect 0.000847 0.000362 0.001677 0.002000 0.000011 -0.000194 0.000772 0.226000

Direct effect 0.005792 0.002689 0.007479 0.000000 0.002703 -0.000719 0.003968 0.170000

Total effect 0.006639 0.003965 0.008387 0.000000 0.002713 -0.000319 0.004269 0.106000

Proportion mediated 0.127621 0.051603 0.340013 0.002000 0.003942 -0.833414 1.195723 0.312000

Unadjusted Analyses: Non-adjusted.

Adjusted Analyses: Adjusted for age, gender, ethnicity, education level, marital status,PIR, Drink, smoking, BMI, Sport,BP,Diabetes.
