# Supplementary material for: Renal function and cognitive performance in older adults: a NHANES-based mediation analysis of methylmalonic acid as a marker of mitochondrial dysfunction
Source: Ren Fail. 2025 Nov 17;47(1):2577843. doi: 10.1080/0886022X.2025.2577843 (PMC12624903; doi:10.1080/0886022X.2025.2577843)
Supplement: Supplementary File 2 R2.docx [file IRNF_A_2577843_SM1667.docx]

**Supplementary File 2** Exploring the association between methylmalonic acid and cognition

**Table S4** Association between methylmalonic acid and cognitive function (CFDAST_z ~ LBXMMASI)

**Standardized β β **95% CI***^1^* **p-value****

Crude Model -0.10235 -0.00069 -0.00108, 0.00031 <0.001

Model1 -0.04432 -0.00030 -0.00062, 0.00001 0.055

Model2 -0.03781 -0.00026 -0.00058, 0.00006 0.104

**Table S5** Association between methylmalonic acid and cognitive function (CFDDS_z ~ LBXMMASI)

**Standardized β β **95% CI***^1^* **p-value****

Crude Model -0.15726 -0.00097 -0.00138, -0.00057 <0.001

Model1 -0.06888 -0.00042 -0.00071, -0.00013 0.007

Model2 -0.06634 -0.00040 -0.00070, 0.00011 0.011

**Table S6** Association between methylmalonic acid and cognitive function (CERAD_z ~ LBXMMASI)

**Standardized β β **95% CI***^1^* **p-value****

Crude Model -0.10592 -0.00068 -0.00101, -0.00034 <0.001

Model1 -0.03728 -0.00023 -0.00050, 0.00003 0.083

Model 2 -0.03263 -0.00021 -0.00048, 0.00007 0.133
