# Supplementary material for: Renal function and cognitive performance in older adults: a NHANES-based mediation analysis of methylmalonic acid as a marker of mitochondrial dysfunction
Source: Ren Fail. 2025 Nov 17;47(1):2577843. doi: 10.1080/0886022X.2025.2577843 (PMC12624903; doi:10.1080/0886022X.2025.2577843)
Supplement: Supplementary FIle 4 R2.docx [file IRNF_A_2577843_SM1666.docx]

**Supplementary File 4** Sensitivity analysis of the association between chronic kidney disease and cognition

|  | **β** | ****95% CI**** | ****p-value**** |
| --- | --- | --- | --- |
| Association between renal function and cognition |  |  |  |
| cognitive_sum | 0.006 | 0.002, 0.010 | 0.008 |
| CFDDS | 0.003 | 0.000, 0.005 | 0.024 |
| CFDAST | 0.001 | -0.001, 0.004 | 0.314 |
| CERAD | 0.002 | -0.001, 0.004 | 0.115 |
| Association between renal function and methylmalonic acid (MMA) | -1.882 | -2.382,-1.381 | ＜0.001 |
| Association between MMA and cognitive function |  |  |  |
| cognitive_sum | -0.001 | -0.002,0.000 | 0.010 |
| CFDDS | 0.000 | -0.001,0.000 | 0.012 |
| CFDAST | 0.000 | -0.001,0.000 | 0.076 |
| CERAD | 0.000 | 0.000,0.000 | 0.138 |

This analysis adjusted for age, sex, race/ethnicity, education level, marital status and poverty-income ratio (PIR), BMI, alcohol consumption, smoking, physical activity, diabetes and hypertension, vitamin B12 level, white blood cell count, lymphocyte percentage, and depression.
